# Severity and disease control before house dust mite immunotherapy initiation: ANTARES a French observational survey

**DOI:** 10.1186/s13223-016-0119-z

**Published:** 2016-04-11

**Authors:** Pascal Demoly, Anne Broué-Chabbert, François Wessel, Antoine Chartier

**Affiliations:** Department of Pulmonology-Division of Allergy, Hôpital Arnaud de Villeneuve, University Hospital of Montpellier, 34295 Montpellier cedex 5, France; Sorbonne Universités, UPMC Paris 06, UMR-S 1136, IPLESP, Equipe EPAR, 75013 Paris, France; Division of Allergy and Immunology, Department of Pediatrics, University Hospital of Toulouse, Toulouse, France; Department of Pneumology, University Hospital of Nantes, Nantes, France; Medical Department, ALK, Courbevoie, France

**Keywords:** Allergen immunotherapy, Allergic rhinitis, House dust mites, Asthma, Pharmaco-epidemiology

## Abstract

**Background:**

Allergen immunotherapy (AIT) may be prescribed for patients with allergic rhinitis (AR) induced by house dust mites (HDM) whether asthma is present or not. Current guidelines provide insufficient support for therapeutic management strategy of these patients. Allergists however have long-term experience with AIT. This study aims to describe the characteristics of the patients seen in clinical practice with HDM allergy and the process used to determine whether AIT should be initiated.

**Methods:**

This was an observational, multicenter, prospective and cross-sectional study, conducted in France from 2013 to 2014 with a representative sample of allergy specialists. Any patient over 5 years of age with confirmed HDM allergy untreated with AIT within the last 12 months was eligible. Data were prospectively collected using physician and patient questionnaires.

**Results:**

A total of 1589 patients (60 % adults, 40 % children) were included by 195 randomly selected allergists. A subgroup of 1212 patients (median age: 22 years; 52 % women) were selected for AIT treatment with a median time of AR diagnosis of 3 years. Amongst these, 59 % had a moderate to severe persistent AR according to AR and its Impact on Asthma guidelines, 57.5 % were polysensitized, and 56.5 % also suffered from conjunctivitis (median rhinitis total symptom score: 11). Asthma was present in 42 % of patients, and was controlled according to Global Initiative for Asthma guidelines in 62 % of patients. The asthma control questionnaire score was 1–1.5 in 20 % and ≥1.5 in 37 % of patients. A total of 57 % patients received a prescription of ≥2 medications (mainly antihistamines). Usual daily activities and sleep quality were slightly-to-moderately impaired as the mean rhinoconjunctivitis quality of life questionnaire score was 2.7 ± 1.5. The major driver of AIT prescription is AR uncontrolled by previous medications leading to patient dissatisfaction.

**Conclusions:**

HDM-AR associated conjunctivitis was present in 60 % and asthma in 40 % of cases. In >40 % of these cases, asthma was inadequately controlled at the start of AIT.

## Background

The prevalence of allergic rhinitis (AR) is increasing and is currently estimated to be 10–20 % of the population worldwide [1]. House dust mites (HDM) are one of the main causes of perennial AR [2]. The prevalence of HDM allergen sensitization varies from 65 to 130 million individuals in the general population worldwide [3]. AR is associated with a high symptom burden and impaired health-related quality of life (HRQoL), sleep [4] and productivity at school or work, with a negative socioeconomic impact caused by absenteeism and passive presenteeism [5–7]. Patients with HDM allergy typically present with symptoms of moderate-to-severe rhinitis. In addition, rhinitis and asthma often coexist in the same patients because AR is associated with allergic asthma (AA) in nearly 50 % of cases [8, 9].

Allergen immunotherapy (AIT) represents a valid therapeutic alternative to HDM avoidance or for patients who experienced symptomatic medication failure or have fears and/or developed side effects to other therapies [10]. In recent years, many randomized, placebo-controlled trials have shown that sublingual immunotherapy (SLIT) is effective at reducing symptoms of AR with a well-tolerated safety profile in children, adults and elderly, which is not optimally controlled by pharmacologic medication and HDM avoidance [11–13]. A meta-analysis also reported a statistically significant reduction in symptoms and medication requirements by AIT compared with placebo [12, 13].

However, the use of AIT in the therapeutic management of both AR and AA needs to be better clarified in everyday practice because current guidelines, especially for asthma [14], do not provide sufficient support for AIT. We need to understand the real life use of AIT and to bridge the gap between real life and guidelines using data from randomized controlled trials. Respiratory allergic diseases should be better phenotyped for AIT indications. The severity level of AA as well as medications and strategies for symptom control and risk reduction were defined in the latest Global Initiative for Asthma (GINA) guidelines [14]. In control-based AA management, pharmacological and non-pharmacological treatments are adjusted in a continuous cycle that involves assessment, treatment and review. For example, the level of AA symptom control depends on the frequency of daytime symptoms, frequency of relief needed for symptom control, night waking caused by disease, and activity limitation [14]. Similarly, the AR and its Impact on Asthma (ARIA) guidelines initiated during a World Health Organization (WHO) workshop in 1999, published in 2001, and last updated in 2010, classified AR as mild/moderate-to-severe and intermittent/persistent [1]. Although this classification underlines the close relationship between rhinitis and AA, physicians are faced with various treatment options for the management of AR [15] and AA separately, contributing to possible variations in clinical practice. Patients with mild AR are unlikely to consult a physician, and therefore, specialists usually see moderate-to-severe AR for which the guidelines are not complete. Moreover, only 62 % of specialists were reported to always or frequently apply the ARIA treatment algorithms in the daily management of AR patients [16]. Other limitations have been described in the literature as these guidelines do not take into account previous and current treatment, they include a heterogeneous group of moderate-to-severe patients, and provide poor guidance on patient management [17]. Therefore, the development of a control-based classification in AR similar to that in the GINA recommendations for asthma would be useful for clinicians [18]. The identification of a particular subgroup of patients for whom AIT initiation is decided by experienced allergists might contribute to the implementation of a stepwise approach.

The present real-life study aims to (1) describe the severity of symptoms, and control levels of AR and AA during the past month before inclusion in a large patient population consulting for HDM-AR and prescribed AIT, (2) identify homogeneous sub-groups by control levels to better guide AIT use in a therapeutic strategy, and (3) describe the benefits experienced by patients with previous symptomatic medication(s) of AR and expectations of AIT.

## Methods

### Study design

This was an epidemiological, observational, multicenter, national, prospective and cross-sectional study. This 3-month study was carried out in France from October 2013 to March 2014. A total of 1600 French allergy specialists (allergists, ear, nose and throat specialists, pediatricians, pulmonologists) randomly selected from a large sample of physicians participated in the study. Each physician was asked to include nine consecutive patients who had been seen for HDM allergy. As AIT is usually initiated in approximately 66 % of cases [19] in French allergist practices, AIT was expected to be prescribed in six out of nine patients. Physicians saw their patients as usual over the course of consultations. The study did not affect patient diagnostic or therapeutic management.

### Ethical considerations

The study was conducted in compliance with the Declaration of Helsinki, Good Epidemiological/Pharmacoepidemiological study guidelines, good practice guidelines and local regulations. All data were collected anonymously. As the study did not fall within the scope of the public health code (Article L1121-1), approvals from Ethics Committees and the French Health Authority (ANSM) were not required.

### Patients

Ambulatory male or female patients, aged at least 5 years, seen in usual consultation with HDM allergy (confirmed by skin testing and/or positive measurement of specific immunoglobulins IgE) with clinical manifestations of AR, able to complete a questionnaire (or by one of the parents or legal representative for minors), informed and willing to participate in the study were selected for inclusion. Patients treated with HDM-AIT within the last 12 months at the time of enrollment were excluded from participation. All patients (or a parent or legal representative) were informed by the physician on the study purpose, and signed an informed consent form before participation.

### Collected data

Data were prospectively collected using a case report form for each patient on the day of the consultation. Patient demographic and clinical characteristics (including medical history, frequency and intensity of AR symptoms according to the ARIA classification, rhinitis total symptom score for sneezing, runny nose, itchy nose, nasal congestion, watery eyes and itchy eyes (0–18 range of scores, the upper value of 18 indicating permanent very severe levels for all six symptoms), comorbidities, and if applicable, stage and level of AA according to the GINA classification were collected. The total nasal symptom score comprised four nasal symptoms (0–12 range). Last available forced expiratory volume in one second (FEV1) value, any drug consumption for AR and AA in the previous 12 months, overall assessment of physician and patient satisfaction towards symptomatic medications were listed. AR and AA control data by current medications with 10 cm visual analog scales (VAS) were confidentially recorded by the physician (0: uncontrolled to 10: controlled).

In addition, a self-report patient questionnaire was completed after consultation. It comprised control testing of AR using a five-item self-assessment AR control test (ARCT) developed for assessing the control of AR [20] (a score of 20 being the cut-off for poor vs well-controlled rhinitis), severity and control level of rhinoconjunctivitis symptoms using a 10-cm VAS, rhinoconjunctivitis quality of life questionnaire (RQLQ) for evaluating AR impact, symptoms of rhinoconjunctivitis (including their severity, and level of control), asthma control questionnaire (ACQ) for asthmatics, benefits (patient benefit questionnaire, PBQ) and expectations (patient needs questionnaire, PNQ) towards concomitant symptomatic medications taken the previous month and the new treatment, respectively [21].

### Statistics

Statistical analysis was performed on the data from all eligible informed patients meeting the inclusion criteria, having filled in a self-administered questionnaire, and receiving a completed prescription at the end of the consultation. This analysis was performed on all patients and by patient age as follows: children (5–17 years) and adults (≥18 years). Categorical variables were compared using the Chi squared test or Fischer’s exact test, and continuous variables were compared using the Student’s *t* test or non-parametric test (Mann–Whitney U-test). Correlations between variables were analyzed using Spearman’s test. Cluster analysis based on classifications and usual scores [22] was performed to develop a useful tool in clinical practice by using nine variables defining demographic and clinical patient characteristics. All statistical hypothesis tests were performed at alpha = 5 % level of significance using SAS^®^ software (version 9.2; SAS^®^ Institute Inc., Cary, NY, USA). No adjustment for multiplicity was performed because this was an observational and exploratory study where data were collected with an objective but not with a pre-specified key hypothesis. The calculation of the number of subjects needed was determined to be 20 % of patients with symptoms of mild AR with an accuracy of 5–6 % in the strata of asthmatic and non-asthmatic patients; no prior assumptions were made. Inferential results of this study cannot be used as evidence but can only be considered as supportive to generate new hypotheses.

## Results

### Patients

A total of 195 specialists included 1589 patients in the study, corresponding to the analysis population. Of these, 1212 patients (76.3 %) received a prescription for AIT. The population consisted of 938 adults and 625 children, with a slight predominance of female adults and male children (Table 1). The mean age was 24 years (range: 4–76 years). Overall, 38.6 % of patients had AR associated with AA. The mean duration of AR since diagnosis was 5.4 ± 7.3 years in patients selected for AIT and 5.3 ± 7.0 years in those not selected.

**Table 1:** Demographic and clinical characteristics at time of consultation

	AIT (N = 1212)	No-AIT (N = 377)	Total (N = 1589)
Age (years)			
Children (N = 625)			
N available	489	136	625
Missing	0	0	0
Mean (SD)	10.7 ± 3.6	11.3 ± 3.6	10.8 ± 3.6
Median	10	12	11
Q1, Q3	8, 14	8, 14	8, 14
Range	4, 17	4, 17	4, 17
Adults (N = 938)			
N available	709	229	938
Missing	0	0	0
Mean (SD)	32.5 ± 10.4	34.0 ± 12.8	32.9 ± 11.1
Median	31	30	31
Q1, Q3	24, 38	24, 41	24, 39
Range	18, 76	18, 74	18, 76
Total (N = 1589)^a^			
N available	1198	365	1563
Missing^a^	14	12	26
Mean (SD)	23.6 ± 13.6	25.5 ± 15.1	24.1 ± 14.0
Median	22	24	22
Q1,Q3	12, 33	14, 35	12, 33
Range	4, 76	4, 74	4, 76
Gender (n, %)			
Children (N = 625)			
N available	488	136	624
Missing	1	0	1
Male	298 (61.1 %)	79 (58.1 %)	377 (60.4 %)
Female	190 (38.9 %)	57 (41.9 %)	247 (39.6 %)
Adults (N = 938)			
N available	709	229	938
Missing	7	7	14
Male	274 (38.6 %)	99 (43.2 %)	373 (39.8 %)
Female	435 (61.4 %)	130 (56.8 %)	565 (60.2 %)
Total (N = 1589)^a^			
N available	1204	370	1574
Missing^a^	8	7	15
Male	575 (47.8 %)	182 (49.2 %)	757 (48.1 %)
Female	629 (52.2 %)	188 (50.8 %)	817 (51.9 %)
Duration of AR since diagnosis (years)			
Children (N = 625)			
N available	451	123	574
Missing	38	13	51
Mean	2.7 ± 2.9	2.6 ± 2.9	2.7 ± 2.9
Median	2.0	1.0	2.0
Q1, Q3	0.5, 4.0	0.5, 4.0	0.5, 4.0
Range	0.0, 16.0	0.0, 12.9	0.0, 16.0
Adults (N = 938)			
N available	678	221	899
Missing	31	8	39
Mean	7.2 ± 8.7	6.9 ± 8.1	7.1 ± 8.5
Median	4.0	3.0	3.0
Q1, Q3	1.0, 10.0	0.8, 10.0	1.0, 10.0
Range	0.0, 55.0	0.0, 40.0	0.0, 55.0
Total (N = 1589)^a^			
N available	1136	353	1489
Missing^a^	76	24	100
Mean	5.4 ± 7.3	5.3 ± 7.0	5.4 ± 7.2
Median	3.0	2.0	2.0
Q1, Q3	0.8, 7.0	0.5, 9.0	0.7, 7.0
Range	0.0, 55.0	0.0, 40.0	0.0, 55.0

^a^The total population corresponding to the analysis population (N = 1589) included patients with missing age (N = 26)

Nearly all patients (97.2 %) were skin prick tested the day of recruitment. HDM specific IgE was measured in 57.7 % of patients, and more frequently in patients who initiated AIT treatment (59 % of adults and 64.4 % of children). A total of 863 patients (56.2 %) were sensitive to at least one other allergen and 673 (43.8 %) were sensitive to HDM allergens only. Other major allergens in polysensitized patients were grass pollen (55.8 %) and dander (43.1 %).

A total of 1187 patients (74.7 % of all included patients, 97.9 % of patients with AIT prescription) were prescribed SLIT. Among patients in the AIT group, 5.9 % were prescribed multiple AIT. Other major allergens given to the patients were grass (8.3 % of adults and 6.1 % of children) and tree pollen (6.1 and 1.9 %, respectively). Overall, the planned maintenance phase was 12 months/year. The daily dose was 300 IR/mL for most patients (98.1 % of adults and 97.1 % of children) and seven times per week for the majority of patients (84.5 and 81.4 %, respectively).

### AR characteristics according to severity (ARIA classification) and control

**Fig. 1:**
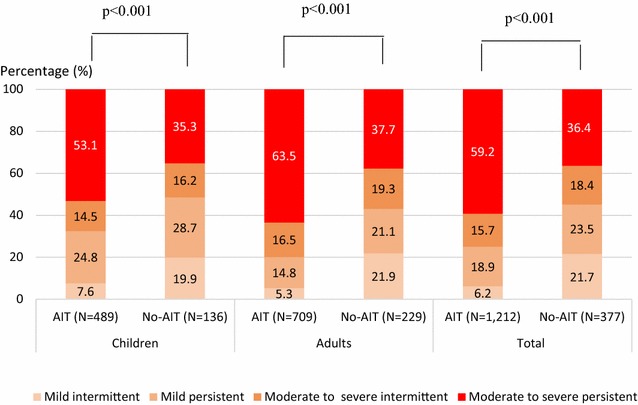
Severity of AR according to the ARIA guidelines. Chi-squared test. *AIT* patients selected for HDM AIT, *No-AIT* patients not selected for AIT

The characteristics of AR according to the ARIA classification are presented in Fig. 1. Overall, moderate-to-severe persistent AR was the most common type of rhinitis (59.2 %). Regardless of patient age, the patients selected for AIT experienced more frequent moderate-to-severe persistent AR than those not selected for AIT (p < 0.001). The most common clinical symptoms of rhinitis considered by the physician to occur very frequently included rhinorrhea (38.2 %), nasal obstruction (37.9 %), and sneezing (37.2 %). Intensity of the most frequent clinical symptoms was mainly moderate. The intensity of AR clinical symptoms assessed by patients using the mean VAS score was significantly higher in patients selected for AIT than those not selected for AIT-treatment (4.6 ± 2.1 vs 4.0 ± 2.1 cm, respectively; p < 0.001). This trend was confirmed for each AR clinical symptom regardless of patient age except for sneezing and nasal obstruction in children for whom the mean VAS score was comparable in both groups. The median rhinitis total symptom score was 11 out of 18 in the group of patients selected for AIT compared with nine in those not selected for AIT (p < 0.001), corresponding to more nasal symptoms (median total nasal symptom score, total nasal symptom score = 8 versus 7 out of 12, respectively; p < 0.001) than ocular symptoms. These symptoms of AR were considered to be moderately-to-severely bothersome in everyday life within the 12 last months of 75.8 % of patients selected for AIT compared to 56.8 % not selected for AIT (p < 0.001). Moreover, according to the results from the RQLQ, patients were more bothered by nasal symptoms (mean score: 3.4 ± 1.4) than ocular symptoms (1.7 ± 1.6). The effect of AR on practical problems and activities/sleep was moderate (mean score: 3.3 ± 1.6 and 2.7 ± 1.5, respectively).

Regarding the level of AR symptom control, the median VAS scores showed that AR was moderately controlled in both groups with a statistically significant difference (4.5 in the group selected for AIT vs 6.2 in the group not selected, p < 0.001). Similarly, the mean ARCT score was significantly higher in the group of patients selected for AIT (17.9 ± 4.0) than in the group not selected (16.7 ± 4.3) (p < 0.001). A moderate correlation was found between both tools, ARCT and VAS for AR control (r = 0.37; p < 0.0001).

### Associated allergy/hypersensitivity conditions

Overall, 1189 patients (76.1 %) had at least one allergy/hypersensitivity co-morbid condition (79.8 % in the AIT group vs 64.3 % in the no-AIT group; p < 0.001). The associated conditions were conjunctivitis (53.8 %) (Table 2), followed by sinusitis and eczema (14.9 % respectively), and urticaria (7.1 %). Conjunctivitis occurred more frequently in patients selected for AIT than other patients (very frequent: 20.9 vs 8.2 %; fairly frequent: 36.5 vs 32.3 %, respectively). The conjunctivitis intensity was moderate and severe in selected AIT-patients (46.9 and 19.7 % respectively), but mild (53.7 %) in patients not selected for AIT.

**Table 2:** Sensitization profile and associated allergic conditions at time of consultation

	AIT (N = 1212)	No-AIT (N = 377)	Total (N = 1589)
Patients with polysensitization (n, %)			
Children (N = 625)			
N available	476	135	611
Missing	13	1	14
n (%)	242 (50.8 %)	73 (54.1 %)	315 (51.6 %)
Adults (N = 938)			
N available	686	222	908
Missing	23	7	30
n (%)	425 (62.0 %)	111 (50.0 %)	536 (59.0 %)
Total (n = 1589)^a^			
N available	1170	366	1536
Missing^a^	42	11	53
n (%)	673 (57.5 %)	190 (51.9 %)	863 (56.2 %)
Patients with asthma (n, %)			
Children (N = 625)			
N available	489	136	625
Missing	0	0	0
n (%)	231 (47.2 %)	48 (35.3 %)	279 (44.6 %)
Adults (N = 938)			
N available	709	229	938
Missing	0	0	0
n (%)	274 (38.6 %)	54 (23.6 %)	328 (35.0 %)
Total (N = 1589)^a^			
N available	1212	377	1589
Missing	0	0	0
n (%)	507 (41.8 %)	106 (28.1 %)	613 (38.6 %)
Patients with conjunctivitis (n, %)			
Children (N = 625)			
N available	477	132	609
Missing	12	4	16
n (%)	259 (54.3 %)	50 (37.9 %)	309 (50.7 %)
Adults (N = 625)			
N available	687	225	912
Missing	22	4	26
n (%)	398 (57.9 %)	108 (48.0 %)	506 (55.5 %)
Total (N = 1589)^a^			
N available	1176	369	1545
Missing	36	8	44
n (%)	664 (56.5 %)	167 (45.3 %)	831 (53.8 %)

^a^The total population corresponding to the analysis population (N = 1589) that included patients with missing age (N = 26)

**Fig. 2:**
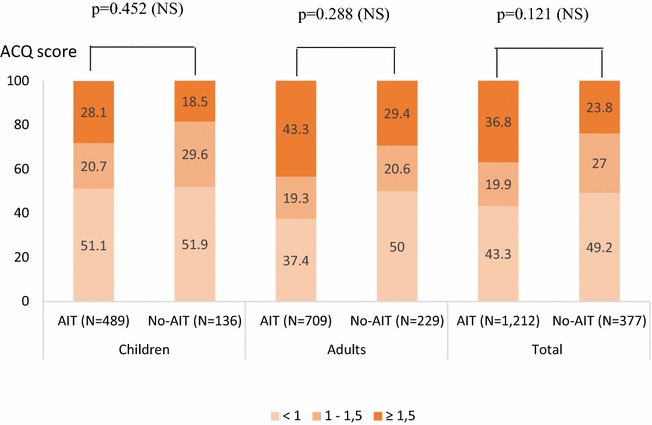
ACQ scores by subgroups. Chi-squared test. *NS* non-significant, *AIT* patients selected for HDM AIT, *No-AIT* patients not selected for AIT

Associated AA was observed in 41.8 % (507/1212) of patients selected for AIT compared with 28.1 % (106/377) of non-selected patients (p < 0.001) (Table 2). FEV1 was ≥80 % in the majority of cases (mean: 84.4 %), varying between 70 and 79 % in 11.7 %, 60–69 % in 3 %, and 50–59 % in 0.9 %. Intensity of AA was persistent mild to moderate in most cases (93 vs 95.1 % in patients selected and not selected for AIT, respectively) according to the GINA classification. In the opinion of the study physicians, AA was considered partly controlled in 32 % in those selected for AIT vs 27.7 % in not selected or was uncontrolled in few cases (6.4 % selected for AIT vs 6.9 % not selected, respectively). The distribution of patients with AA in treatment steps 1, 2, 3, 4, and 5 was 34.7, 26.9, 31.4, 6.7 and 0.2 % of patients who started AIT, respectively, with no marked differences compared with patients not selected for AIT (31.1, 35.0, 29.1, 2.9 and 1.9 %, respectively) (Table 3). However, there were more asthmatics in the group selected for AIT treatment who were reported to be uncontrolled by the ACQ questionnaire (ACQ ≥1.5: 36.8 % in the group selected for AIT vs 23.8 % in the group not selected for AIT, respectively) but this difference was not statistically significant (Fig. 2). The VAS score for AA control decreased in parallel with more uncontrolled AA (Table 3) with a significant correlation between both VAS and ACQ scores (Spearman ratio = −0.535; p < 0.0001). The level of AA symptom control was comparable in both groups as shown by the mean ACQ scores (1.3 ± 0.9 in the group selected for AIT vs 1.2 ± 1.0 in the group not selected for AIT; p = non-significant [NS]) and the mean VAS scores (7.5 ± 2.7 vs 7.6 ± 2.7; p = NS).

**Table 3:** Control of asthma assessed by the physician using VAS, ACQ score and GINA classification

ACQ	<1 N = 164	1–1.5 N = 78	≥1.5 N = 128	p value**
Control of asthma (VAS)				
N available	164	78	128	
Mean ± SD	8.64 ± 1.79	7.41 ± 2.63	6.10 ± 2.67	<0.001
Median	9.4	8.3	6.5	
Min, Max	1.8, 10.0	0.8, 10.0	0.6, 10.0	

GINA classification	Controlled N = 366	Partly controlled N = 184	Uncontrolled N = 38	p value**
Control of asthma (VAS)				
N available	366	184	38	
Mean ± SD	8.8 ± 1.8	5.8 ± 2.0	2.5 ± 2.2	<0.001
Median	9.4	6.2	1.8	
Min., Max	0.0, 10.0	0.8, 9.3	0.0, 8.4	

Treatment steps according to GINA classification	AIT	No-AIT	Total	p value**
Children (N = 279)				
N available	224	46	270	
Missing	7	2	9	
Step 1	67 (29.9 %)	13 (28.3 %)	80 (29.6 %)	0.102
Step 2	70 (31.3 %)	15 (32.6 %)	85 (31.5 %)	
Step 3	73 (32.6 %)	15 (32.6 %)	88 (32.6 %)	
Step 4	14 (6.3 %)	1 (2.2 %)	15 (5.6 %)	
Step 5	0 (0.0 %)	2 (4.3 %)	2 (0.7 %)	
N available	224	46	270	
Adults (N = 328)				
N available	265	53	318	
Missing	9	1	10	
Step 1	103 (38.9 %)	18 (34.0 %)	121 (38.1 %)	0.502
Step 2	61 (23.0 %)	18 (34.0 %)	79 (24.8 %)	
Step 3	81 (30.6 %)	15 (28.3 %)	96 (30.2 %)	
Step 4	19 (7.2 %)	2 (3.8 %)	21 (6.6 %)	
Step 5	1 (0.4 %)	0 (0.0 %)	1 (0.3 %)	
Total (N = 613)^a^				
N available	490	103	593	
Missing	17	3	20	
Step 1	170 (34.7 %)	32 (31.1 %)	202 (34.1 %)	0.066
Step 2	132 (26.9 %)	36 (35.0 %)	168 (28.3 %)	
Step 3	154 (31.4 %)	30 (29.1 %)	184 (31.0 %)	
Step 4	33 (6.7 %)	3 (2.9 %)	36 (6.1 %)	
Step 5	1 (0.2 %)	2 (1.9 %)	3 (0.5 %)	

** Kruskal–Wallis test or Fisher’s exact test

^a^The total population corresponding to the whole population of asthmatics (N = 613) that included patients with missing age (N = 6)

### Previous and concomitant symptomatic medications

**Fig. 3:**
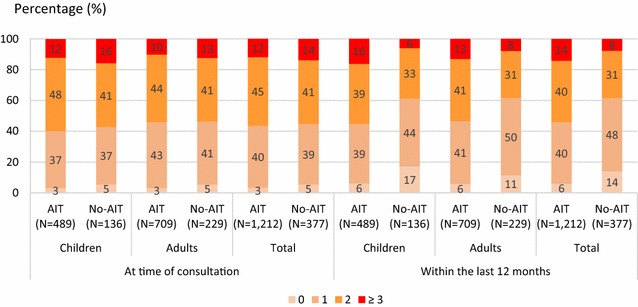
Number of symptomatic medications received at time of consultation and within the last 12 months. *AIT* patients selected for HDM AIT, *No-AIT* patients not selected for AIT

**Fig. 4:**
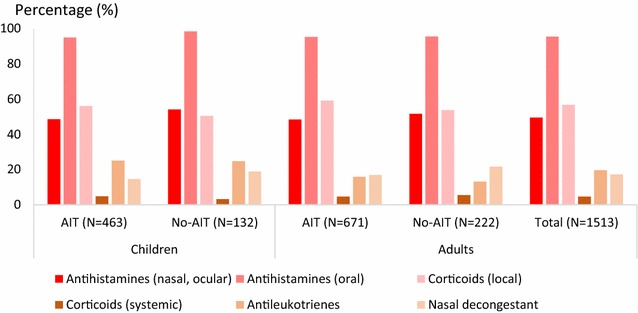
Associated symptomatic medications by therapeutic classes at time of consultation. *AIT* patients selected for HDM AIT. *No-AIT* patients not selected for AIT

Within the last 12 months, monotherapy for AR was received by 41.8 % of patients, bitherapy in 37.8 % and ≥3 medications in 12.7 % (Fig. 3). At the time of HDM-AIT initiation, 1497 patients (98.9 %) were prescribed symptomatic medications: 39.6 % of all included patients were treated with monotherapy, and 42.1 and 13.4 % received 2 or ≥3 symptomatic medications, respectively. The number of symptomatic medications received was similar irrespective of the patient age and selected group (AIT vs no-AIT) (Fig. 3). The main prescribed symptomatic medications were oral antihistamines (95.5 %) (Fig. 4). Treatment with systemic corticoids was uncommon (≤5 % of patients). Nearly all AA patients (97.7 %) received at least one medication for AA. At the time of AIT initiation, prescriptions with short acting beta-agonists (SABA) were renewed in 81.6 % in patients with AA. New prescriptions with SABA (7.3 %) and/or long acting beta-agonists (3.9 %) were limited. The same was observed for oral and inhaled corticoids, and for anti-leukotrienes.

### Physician and patient expectations and satisfaction of previous medications

Regardless of the group of patients, 94.5 % of physicians reported they would like an improvement of AR symptoms and quality of life (QoL). The main expectations stated by about 90 % of patients that were considered to be significant to very significant towards their new prescribed treatments were the relief of symptoms, no nasal congestion or runny nose, ease of breathing through the nose, and no sneezing fits, regardless of the group asked (Table 4). Overall, these expectations were significantly more frequent in patients selected for AIT than others. More than 75 % of patients highlighted an interest in the ease of use with a statistically significant difference between both groups: 84.7 % in the group selected for AIT vs 76.9 % in the group not selected (p = 0.006).

**Table 4:** Patient needs at time of consultation

	Percentage of quite/very important responses^a^			p value**
	AIT (N = 1083), %	No-AIT (N = 335), %	Total (N = 1418**),** %	
To be relieved of all symptoms	96.1	92.4	95.2	0.020
To no longer have a runny or blocked nose	94.9	92.2	94.3	0.179
To be able to breathe through my nose more freely	92.1	89.4	91.5	0.296
To not have sneezing impulses	87.6	83.9	86.7	0.176
To have a treatment which is easy to use	84.2	76.9	82.5	0.006
To not experience eye, nose or palate stinging anymore	82.0	73.8	80.1	0.003
To have confidence in the therapy	82.8	70.2	79.9	<0.001
To experience more enjoyment of life	76.7	66.0	74.2	<0.001
To be able to sleep better	75.2	62.4	72.2	<0.001
To be able to concentrate better at work	73.1	66.0	71.4	0.042
To be able to stay outdoors without symptoms	72.8	66.7	71.3	0.033
To be able to engage in normal leisure activities	73.9	61.7	71.0	<0.001
To have no fear that the disease will become worse	72.4	61.2	69.7	0.001
To feel less tired or groggy	71.8	62.6	69.6	<0.001
To be more productive in everyday life	69.6	60.6	67.5	0.009
To not have burning or watery eyes anymore	67.6	57.2	65.2	0.002
To reduce the frequency of visits to the physician	68.3	54.3	65.0	<0.001
To feel more comfortable in public	58.4	54.2	57.4	0.229
To feel less burdened in your relationship	59.2	51.7	57.4	0.057
To spend less time on daily treatment	57.5	51.1	56.0	<0.001
To have fewer side effects	55.5	46.7	53.5	0.019
To feel less irritated	54.7	44.8	52.3	0.007
To have fewer out-of-pocket treatment expenses	53.	46.1	51.6	0.012
To feel less depressed	44.9	35.8	42.8	0.002
To be able to have a normal sex life	41.5	38.6	40.8	0.532

Analysis of the patient needs questionnaires (PNQ) completed on the day of the consultation, corresponds to the needs of the patient (i.e. what he/she expected from a new therapeutic management of disease by her/his physician). Patient needs are ordered by decreasing importance in the total population. Needs are rated using a six-point scale from ‘not concerned’ to ‘very important’

** Chi squared test or Fisher’s exact test

^a^Percentage of patients with needs corresponding to ‘quite important’ to ‘very important’ at allergen immunotherapy initiation

Over 33 % of patients considered that symptomatic medications for AR used during the last month were not helpful for almost all factors described in the PBQ such as leading a normal sexual life, feeling less depressed, less irritable, or less tired/groggy (Table 5). However, there were some exceptions because 62 % of patients judged that medications had a somewhat to very important support in relieving symptoms.

**Table 5:** Patient benefits related to medications for AR taken during the past month at time of consultation

	Percentage of patients helped rather/a lot^a^			p value**
	AIT (N = 1012), %	No-AIT (N = 301), %	Total (N = 1313), %	
To have a treatment which is easy to use	71.6	69.8	71.2	0.541
To have confidence in the therapy	62.9	61.4	62.6	0.635
To be relieved of all symptoms	60.3	67.2	61.9	0.031
To not have sneezing impulses	58.3	62.2	59.2	0.230
To be able to breathe through my nose more freely	55.8	66.2	58.2	0.001
To no longer have a runny or blocked nose	56.5	63.0	58.0	0.049
To experience more enjoyment of life	58.3	53.9	57.3	0.188
To be able to engage in normal leisure activities	57.5	53.4	56.6	0.219
To not experience eye, nose or palate stinging anymore	53.5	56.6	54.2	0.361
To be able to stay outdoors without symptoms	52.8	56.5	53.6	0.264
To be able to sleep better	53.5	48.8	52.5	0.156
To reduce the frequency of visits to the physician	50.4	48.4	49.9	0.566
To be able to concentrate better at work	49.3	50.3	49.6	0.763
To have no fear that the disease will become worse	49.2	49.0	49.1	0.950
To be more productive in everyday life	49.1	46.5	48.5	0.447
To not have burning or watery eyes anymore	48.3	44.7	47.5	0.285
To have fewer side effects	46.9	42.7	45.9	0.207
To feel more comfortable in public	45.3	46.1	45.5	0.818
To feel less burdened in your relationship	44.2	45.9	44.6	0.622
To spend less time on daily treatment	42.8	45.9	43.5	0.357
To feel less tired or groggy	42.6	41.8	42.4	0.804
To have fewer out-of-pocket treatment expenses	42.6	35.5	41.0	0.036
To feel less irritated	40.0	36.2	39.1	0.252
To feel less depressed	35.4	34.3	35.1	0.741
To be able to have a normal sex life	32.8	35.3	33.4	0.503

Analysis of the patient benefits questionnaires (PBQ) completed the day of the consultation correspond to the benefits that the patient expressed relative to the previous symptomatic therapeutic management for AR. Patient benefits are ordered by decreasing importance of the corresponding need. Treatment-related benefits are rated using a five-point scale from ‘did not help at all’ to ‘helped a lot’

** Chi squared test

^a^Percentage of patients with benefits achieved by treatments for AR from ‘rather helped’ to ‘helped a lot’ at allergen immunotherapy initiation

### Cluster analysis

A cluster analysis was performed on 1456 patients with AR having completed the questionnaires and without major deviation to the protocol to understand the allergists’ criteria to prescribe AIT.

Three clusters with statistically significant differences were identified: age, sex, frequency of associated AA, AR severity and level of control, ARCT score, physician/patient satisfaction rate, and prescription of AIT (Table 6). Patients in cluster 1 had moderate and partly controlled AR. The majority of patients in cluster 2 experienced mild and well controlled AR, while those in cluster 3 who were mostly females (61.6 %) and older than in other clusters (median age: 27 years) had very severe and uncontrolled AR. AIT was more often prescribed in patients belonging to cluster 3 (82.8 %) compared to clusters 1 and 2 (78.6 and 61.8 % respectively, p < 0.001).

**Table 6:** Characteristics of the different clusters identified

Cluster	1 N = 854	2 N = 306	3 N = 296	p value
Gender (n, %)				
N available	847	303	292	
Missing	7	3	4	
Male	408 (48.2 %)	167 (55.1 %)	112 (38.4 %)	<0.001*
Female	439 (51.8 %)	136 (44.9 %)	180 (61.6 %)	
Age (years)				
N available	840	301	292	
Missing	14	5	4	
Mean ± SD	23.9 ± 13.8	22.0 ± 14.8	26.5 ± 13.4	<0.001**
Median	22	18	27	
Q1, Q3	12, 33	10, 30	16, 34	
Range	4, 75	5, 70	5, 76	
Allergic asthma				
N available	854	306	296	
No	549 (64.3 %)	171 (55.9 %)	179 (60.5 %)	0.030*
Yes	305 (35.7 %)	135 (44.1 %)	117 (39.5 %)	
Severity of AR (ARIA)				
N available	843	305	294	
Missing	11	1	2	
Mild intermittent/persistent	202 (24.0 %)	216 (70.8 %)	16 (5.4 %)	<0.001*
Moderate to severe intermittent	169 (20.0 %)	32 (10.5 %)	32 (10.9 %)	
Moderate to severe persistent	472 (56.0 %)	57 (18.7 %)	246 (83.7 %)	
Levels of AR control (VAS)				
N available	806	285	280	
Missing	48	21	16	
Mean ± SD	4.48 ± 2.67	7.01 ± 2.59	3.76 ± 2.81	<0.001**
Median	4.2	7.8	2.9	
Q1, Q3	2.3, 6.8	5.6, 9.0	1.5, 6.0	
Range	0.0, 10.0	0.0, 10.0	0.0, 10.0	
Physician satisfaction				
N available	766	266	261	
Missing	88	40	35	
Dissatisfied/very dissatisfied	493 (64.4 %)	51 (19.2 %)	193 (73.9 %)	<0.001*
Satisfied/very satisfied	273 (35.6 %)	215 (80.8 %)	68 (26.1 %)	
Patient satisfaction				
N available	808	292	280	<0.001*
Missing	46	14	16	
Dissatisfied/very dissatisfied	463 (57.3 %)	51 (17.5 %)	200 (71.4 %)	
Satisfied/very satisfied	345 (42.7 %)	241 (82.5 %)	80 (28.6 %)	
ARCT score				
N available	816	294	281	
Missing	38	12	15	
Mean ± SD	16.6 ± 3.5	21.1 ± 2.8	13.7 ± 4.0	<0.001**
Median	17	21	13	
Q1, Q3	14, 19	20, 23	11, 16	
Range	5, 25	10, 25	5, 25	
Prescription of AIT				
N available	854	306	296	
No	183 (21.4 %)	117 (38.2 %)	51 (17.2 %)	<0.001*
Yes	671 (78.6 %)	189 (61.8 %)	245 (82.8 %)	

* Chi squared test, ** Kruskal–Wallis test

## Discussion

The findings of this study reveal the profile of patients being initiated on HDM AIT-treatment in allergist practices in France. The major driver of AIT prescription is an AR uncontrolled by previous medications leading to patient dissatisfaction. At treatment initiation, patients were young. The management takes into account comorbidities, such as AA and rhinoconjunctivitis [23]. Our results were consistent with a previous study [24] showing that more children than adults experience AR with AA (44.6 vs 35 %). Moreover, the occurrence of AA doubled the likelihood of selecting for HDM AIT treatment in our study. The results suggest that the severity and level of control differed between patients selected or not for AIT treatment. Of note, AA was insufficiently controlled in approximately 33 % of patients before starting AIT, and the QoL was particularly impacted in patients selected for AIT. Uncontrolled AA does not limit AIT prescription (38.4 % of inadequately controlled patients in the group selected for AIT), probably because it was reported that AIT is effective in the treatment of AR and asthma for HDM sensitivity [25]. Therefore, the mutual evaluation of AR and AA control is needed before prescribing an appropriate treatment.

HDM AIT solution was prescribed in almost all patients with a sublingual concentration of 300 IR/mL every day, which was reported to have the best benefit-risk profile in clinical practice [26]. Non-compliance to the treatment may have a strong impact on the condition of patients suffering from chronic disease. A systematic review of publications that assessed adherence showed that 55–82 % of patients discontinued SLIT before completing the recommended treatment duration of 3 years [27, 28]. The main causes of non-adherence may be linked to lower socioeconomic status, younger age [28], and cost of treatment [29]. However, one study that attempted to test an educational intervention with a strict follow-up showed a significant improvement in SLIT adherence [30]. Concerning patient satisfaction, about 66 % of patients highlighted the importance of a therapy for AR in relieving symptoms regardless of the patient group and the ease of use. Indeed, SLIT has been shown to be effective in adults and pediatric populations with HDM allergy across several studies [24, 31–33]. Moreover, use of SLIT is easy, non-invasive, painless especially important for children, and adapted for administration at home [34].

Our survey has some limitations as its design was non-interventional and observational. A potential bias may be found in the selection of patients and results. However, the strengths of the study conducted in a real-life setting with allergy specialists are the large size of the analysis population of patients (n = 1589) that may be considered the most representative of those suffering from HDM allergy and AR in France. This large number of patients has enabled the composition of both groups, those starting HDM AIT and those selected for other therapy, to be stratified by age. Moreover, the data collection used reproducible, standardized and validated questionnaires. Finally, this study allowed us to differentiate separate rhinitis clusters by sex, frequency of AR symptoms, prescriptions of AIT and symptomatic medications, rhinitis severity and QoL. This classification may be an additional helpful tool for the physician as a complement to severity-based approach using ARIA guidelines. In our study, the most severe cluster that merits attention was associated with young age of onset (median: 27 years), female sex, with frequent and severe nasal and ocular symptoms, impaired QoL and frequent prescriptions of AIT.

## Conclusions

Our study conducted in a real-life setting identified a population of patients affected by HDM-AR for at least 2 years and seen by allergy specialists. The patients selected for HDM AIT treatment were characterized by the presence of conjunctivitis and asthma. At the time of AIT prescription, over 40 % of patients already experienced inadequately controlled asthma, and about 60 % had mild to severe persistent AR. The major driver of AIT prescription was an AR uncontrolled by previous medications leading to patient dissatisfaction.
